# Obstetric Outcomes among Syrian Refugees: A Comparative Study at a Tertiary Care Maternity Hospital in Turkey

**DOI:** 10.1055/s-0038-1673427

**Published:** 2018-10-11

**Authors:** Sule Ozel, Selen Yaman, Hatice Kansu-Celik, Necati Hancerliogullari, Nurgul Balci, Yaprak Engin-Ustun

**Affiliations:** 1 Department of Obstetrics and Gynecology, University of Health Science, Zekai Tahir Burak Woman's Health, Education and Research Hospital, Ankara, Turkey; 2 Ministry of Health Turkish Public Hospitals Institution Association, Family and Community Medicine, Ankara, Turkey

**Keywords:** Syrian Refugees, immigrants, obstetric outcomes, neonatal outcomes, antenatal care

## Abstract

**Objective** The aim of this study was to analyze and compare obstetric and neonatal outcomes between Syrian refugees and ethnic Turkish women.

**Methods** Retrospective, observational study. A total of 576 Syrian refugees and 576 ethnic Turkish women were included in this study, which was conducted between January 2015 and December 2015 at a tertiary maternity training hospital in Ankara, Turkey. The demographic characteristics, obstetric and neonatal outcomes were compared. The primary outcomes were pregnancy outcomes and cesarean rates between the groups

**Results** The mean age was significantly lower in the refugee group (*p* < 0.001). Mean gravidity, proportion of adolescent pregnancies, proportion of pregnant women aged 12 to 19 years, and number of pregnancies at < 18 years were significantly higher among the refugee women (*p* < 0.001). Rates of antenatal follow-up, double testing, triple testing, gestational diabetes mellitus (GDM) screening, and iron replacement therapy were significantly lower in the refugee group (*p* < 0.001). The primary Cesarean section rate was significantly lower in the refugee group (*p* = 0.034). Pregnancies in the refugee group were more complicated, with higher rates of preterm delivery (< 37 weeks), preterm premature rupture of membranes (PPROM), and low birth weight (< 2,500 g) when compared with the control group (4.2% versus 0.7%, *p* < 0.001; 1.6% versus 0.2%, *p* = 0.011; and 12% versus 5.8%, *p* < 0.001, respectively). Low education level (odds ratio [OR] = 1.7, 95% confidence interval [CI] = 0.5–0.1), and weight gain during pregnancy (OR = 1.7, 95% CI = 0.5–0.1) were found to be significant indicators for preterm birth/PPROM and low birthweight.

**Conclusion** Syrian refugees had increased risks of certain adverse obstetric outcomes, including preterm delivery, PPROM, lower birth weight, and anemia. Several factors may influence these findings; thus, refugee women would benefit from more targeted care during pregnancy and childbirth.

## Introduction

People moving out of their country due to restriction or danger to their lives are vulnerable. Syrian refugees are citizens of Syria who have fled their country since the onset of the Syrian civil war, in 2011. Because of its geographical location and politics, Turkey has been the first choice of Syrian asylum seekers.^1^ International temporary protection was an urgently granted temporary status decided on by the Council of Turkish Ministers when this massive migratory movement occurred. Temporary protection regulations were enacted on October 22, 2014. According to these regulations, healthcare must be given to Syrian asylum seekers in their province of residence in Turkey.^2^

There are currently 2,973,980 registered Syrian refugees in Turkey; 23.6% of them are females over 18 years of age.^3^ The proportion of Syrian refugees among the population in Ankara is 1.32%.^2^ One objective of the Healthy People 2020 Project is to increase the proportion of pregnant women who receive early and adequate prenatal care.^4^ Pregnant refugee women face multiple and complex systematic obstacles to perinatal care, such as language and cultural barriers, lack of resources, difficulty navigating a complex system and diverse migratory experience and legal status.^5^ Timely care of pregnant refugees can decrease their vulnerability. The goal of prenatal care for all pregnant refugees is the provision of qualified obstetrical care, which contributes to a safe birth and favorable outcomes for both the mother and child. Without this, obstetric complications may occur, which require lengthy recovery times, such that female refugees may face lasting physical, psychological, and economic consequences. In Turkey, Syrian refugees have free access to primary and secondary healthcare facilities in public hospitals. Of all women giving birth at our Tertiary Care Maternity Hospital in 2015, 3.2% were refugees; they were at higher risk for certain adverse obstetric outcomes.

The aim of this study was to analyze and compare obstetric and neonatal outcomes between Syrian refugees and ethnic Turkish women giving birth at a maternity clinic in Ankara, Turkey.

## Methods

This was a retrospective cohort study performed between January 2015 and December 2015 at Zekai Tahir Burak Woman's Health, Education and Research Hospital, a tertiary government maternity training hospital located in Ankara, the capital city of Turkey. The study protocol was approved by the institutional review board of the hospital (No. 30/2015, December 2015). During this study period, the first ethnic Turkish women to give birth after each Syrian refugee woman (using the protocol number) who delivered at our hospital were enrolled in this study. The primary outcomes were pregnancy outcomes and cesarean rates between the groups. Maternal age, body mass index (BMI), obstetric history, education level, number of antenatal follow-ups, hemoglobin and hematocrit levels, clinical findings, and obstetric and neonatal outcomes were obtained from medical records. Data on maternal age, rate of adolescent pregnancy, weight gain during pregnancy (WGPD), weight gain per week, educational level, number of antenatal follow-ups, route of delivery, primary Cesarean section (C-section) rate, induction of labor, meconium-stained amniotic fluid, amount of red cell transfusion, and obstetric complications (including preterm delivery, postterm pregnancy (> 41 weeks gestational age), preeclampsia, stillbirth, macrosomia (birth weight > 4,000 g), premature rupture of membranes (PROM), preterm premature rupture of membranes (PPROM), and postpartum hemorrhage were obtained through medical chart review. Hematocrit and hemoglobin levels were evaluated at admission for delivery. Education level was classified according to the number of years spent at primary and secondary school, high school and university (1: uneducated, 2: 0 to 8 years, 3: > 8 years). Neonatal outcomes, including birth weight, small for gestational age (SGA), low birth weight (< 2,500 g) and height, head circumference, fetal gender, first and fifth minute Apgar scores, and neonatal intensive care unit (NICU) admission were also obtained from medical records. Type of delivery was noted as vaginal or Cesarean. Indications for Cesarean delivery included previous history of C-section, fetal distress, cephalopelvic disproportion, breech presentation, and others.

The estimation of gestational age was based on the last menstrual period and fetal crown-rump-length on the basis of ultrasound performed between 11 and 14 weeks of gestation. BMI was defined as pre-pregnancy weight in kilograms divided by height in meters squared and categorized three group as BMI < 19, 19 to 25, and > 25. Weight gain during pregnancy is defined as the amount of weight gained between before conception and just before the birth of the infant. Weight gain per week calculated as WGDP divided (g) gestational week at birth. Preterm delivery was defined as delivery before 37 completed gestational weeks,^6^ and PROM referred to rupture of membranes before the onset of uterine contractions; PPROM referred to PROM before 37 weeks of gestation.^7^ Preeclampsia was described as new-onset hypertension (> 140/90 mm Hg) after 20 weeks of gestation in a previously normotensive woman, accompanied by proteinuria (> 300 mg/24 hour).^8^ Small for gestational age was defined as infants with a birth weight below the 10^th^ percentile for gestational age.^9^ Stillbirth was described as a baby born with no sign of life at or after 28 weeks' gestation, according to the World Health Organization (WHO) criteria.^10^ All of the subjects underwent a two-step gestational diabetes mellitus (GDM) screening between 24 and 28 weeks of gestation. A positive 50-g glucose challenge test (GCT) is defined as a glucose level one hour after glucose challenge of at least 140 mg/dl. Women who had a positive 50-g GCT were advised to follow a normal diet 3 days before the 100-g oral glucose tolerance test (OGTT). Gestational diabetes mellitus was diagnosed if there were two or more abnormal values with a 100 g OGTT performed according to the criteria identified by Carpenter and Coustan.^11^ The double test at first trimester (using ultrasound measurement of nuchal translucency, pregnancy-associated plasma protein A, and human chorionic gonadotrophin [hCG]) and triple test at second trimester (using α-fetoprotein, hCG, and uE3) were conducted to identify whether their fetus has an aneuploidy.^12^ Emergency birth was defined as a birth that occurs accidentally or precipitously in the hospital without standard obstetric preparation and procedures. Neonatal death was defined as death occurring within the first 27 completed days of life.

Statistical analyses were performed using the SPSS software ver. 17.0 (SPSS Inc., Chicago, IL, USA). Means and standard deviations (SDs) were calculated for continuous variables. Subject characteristics and demographics were analyzed descriptively. The normality of the distribution of the variables was analyzed by the Kolmogorov-Smirnov test. The Student *t*-test, the Mann-Whitney U test, and the χ^2^ test or Fisher exact test were used to evaluate associations between categorical and continuous variables. Odds ratios and 95% confidence intervals (CIs) for preterm birth, PPROM, and low birthweight were calculated by a logistic regression model. Two-sided *p*-values were considered statistically significant at *p* < 0.05.

Primary outcome of this study is to compare the obstetric outcomes of two groups. The assumption to estimate the sample size was based on Preterm delivery (< 37 weeks). After sorting through the patient files the primary data was yielded and power analysis conducted for the calculation of the sample size. Sample sizes of 576 and 576 for each group were detected, with a 98.6% power at a significance level (α) of 0.05.

## Results

There were 17,704 births at Zekai Tahir Burak Woman's Health, Education and Research Hospital during the study period. Of these, 3.2% of the mothers were Syrian refugees. In total, 576 Syrian refugees and 576 pregnant ethnic Turkish women were included in this study. Thus, in total, the births of 1,152 women were analyzed. Demographic and medical characteristics of the patients are shown in Table 1. Mean age, gestational age at delivery, WGPD, and weight gain per week were all significantly lower in the refugee group (*p* < 0.001). Body mass index > 25 kg/m^2^ was more common in the refugee group than in the control group (412 (82%) versus 436 (75%); *p* = 0.006). The proportions of adolescent pregnancies aged 12 to 19 years, and pregnancies < 18 years were significantly higher among the Syrian refugees (*p* < 0.001). The mean gravidity of the Syrian refugees was significantly higher than that of the Turkish women (*p* < 0.001). Parity > 3 in the refugee group was more common than in the control group (130 [22%] versus 48 [8%]; *p* < 0.001). Hemoglobin and hematocrit levels were significantly lower in the Syrian refugees than in the Turkish women (*p* < 0.001). The proportion of uneducated women was significantly higher in the refugee group (*p* < 0.001). There were no statistically significant differences between groups in terms of the rates of abortion or dilatation and curettage (D&C) (p > 0.05).

**Table 1:** Demographics and medical history

Variables	Refugee Group	Control groups	*p*-value
	(*n* = 576)	(*n* = 576)	
Age (years) median (min–max)	23 (14–44)	27 (16–44)	< 0.001*
Adolescent pregnancy (< 21 years) (n/%)	183 (31%)	54 (10%)	< 0.001*
Pregnancy with < 19 years (n/%)	57 (%10)	5 (1%)	< 0.001*
Gravida (mean ± SD)	2.92 ± 2(1–15)	2.27 ± 1.3 (1–9)	< 0.001*
Parity (n/%)			< 0.001*
Nullipara	200 (34%)	233 (40%)	
1	246 (42%)	295 (51%)	
≥ 2	130 (22%)*	48 (8%)*	
Previous abortion median (min–max)	0 (0–5)	0 (0–7)	0.288
Previous D&C median (min–max)	0 (0–4)	0 (0–5)	0.114
Maternal BMI n (%)			0.006*
< 19	8 (1.4%)	4 (0.8%)	
19–25	132 (23%)*	83 (16%)	
> 25	436 (75%)	412 (82%)*	
Gestational age(weeks) (mean ± SD)	37.49 ± 2.05	38.14 ± 1.78	< 0.001*
WGDP (kg) (mean ± SD)	9.33 ± 4.28	11.87 ± 4.59	< 0.001*
Weight gain per week (g)	25.29 ± 11.6	31.06 ± 11.7	< 0.001*
Education level (n/%)			< 0.001*
1	308 (53%)*	11 (1.9%)*	
2	133 (23%)*	432 (75%)*	
3	51 (8.9%)*	20 (3.5%)*	
Maternal systemic disease (n/%)	17 (3%)	17 (3)	1
Hb levels (mg/dl) median (min–max)	11.7 (6.1–15)	12 (7.7–14.7)	< 0.001*
Anemia (Hb < 12 mg/dl) (n/%)	332 (57%)	197 (39%)	< 0.001*
Hematocrit median (min–max)	35 (22–45)	36 (24–45)	< 0.001*

Abbreviations: BMI, body mass index; D&C, dilatation and curettage; Hb, hemoglobin; SD, standard deviation; WDGP, weight gain during pregnancy.

Education level = 1: uneducated, 2: 0–12 years, 3: >12 years; Maternal systemic disease included diabetes and hypertension.

* *p* < 0.05, significant.

A comparison of antenatal parameters is shown in Table 2. The rates of antenatal follow-up, double test, triple test, gestational diabetes mellitus screening test and iron replacement therapy were significantly lower in the refugee group (*p* < 0.001).

**Table 2:** Antenatal parameters

Variable n (%)	Refugee group	Control groups	*p*-value
	(*n* = 576)	(*n* = 576)	
Antenatal follow-up	137 (23%)	497 (86%)	< 0.001*
Double test	92 (16)	465 (80%)	< 0.001*
Triple test	92 (16)	470 (81%)	< 0.001*
GDM screening test	88 (15%)	464 (80%)	< 0.001*
Antenatal iron supplementation	301 (52%)	450 (78%)	< 0.001 *

Abbreviation: GDM, Gestational diabetes mellitus.

* *p* < 0.05, significant.

Tables 3 and 4 summarize the obstetric and neonatal outcomes of the Syrian women. There was no statistically significant difference between the groups in terms of delivery type, indications for C-section, amount of red cell transfusion, number of congenital abnormalities, postterm pregnancy, stillbirth, preeclampsia, PROM, SGA, NICU admission, neonatal death, or fetal gender (*p* > 0.05). Emergency births, the requirement for labor induction using oxytocin, and meconium-stained amnions were more common in the refugee group (*p* < 0.001). The primary C-section rate was significantly lower in the refugee group (*p* = 0.034). Pregnancies in the refugee group were more complicated, with higher rates of preterm delivery (< 37 weeks), PPROM, and low birth weight (< 2,500 g) versus the control group (*p* < 0.001, *p* = 0.011, and *p* < 0.001, respectively). Macrosomia (birth weight > 4,000 g) was more common in the control group (*p* = 0.03). Birth weight, height, and head circumference were lower in the refugee group (*p* < 0.001). Rate of newborn Apgar scores < 7 at 1 and 5 minutes were significantly higher in the refugee group (*p* < 0.001).

**Table 3:** Obstetric outcomes

Variable n (%)	Refugee group	Control groups	*p*-value
	(*n* = 576)	(*n* = 576)	
Emergency birth	121 (21%)	23 (4%)	< 0.001*
Mode of delivery			0.084
Vaginal birth	391 (67%)	355 (51%)	
C-Section	185 (32%)	221 (38%)	
Primary C-section rate	94 (16%)	107 (21%)	0.034*
Indications of C-section			0.068
Previous C-section	91 (15%)	95 (16%)	
Fetal distress	39 (6%)	51 (9%)	
Cephalopelvic disproportion	13 (2)	30 9(5%)	
Breech presentation	9 (1%)	14 (2%)	
Others	33 (5%)	31 (5%)	
Requirement induction of labor	107 (18%)	217 (37%)	< 0.001*
Type of induction			< 0.001*
Oxytocin	104 (18%)*	170 (29%)	
Dinoprostone vaginally	4 (0.7%)	8 (1.4%)	
Meconium stained amnions	46 (8%)	13 (2.3%)	< 0.001*
Amount of red cell transfusion	2 (1–5)	2 (1–2)	1
Congenital abnormality	3 (0.5%)	1 (0.2%)	0.135
Preterm delivery (< 37 weeks)	24 (4.2%)	4 (0.7%)	< 0.001*
Postterm pregnancy (> 41 weeks)	17 (3%)	15 (2.6%)	0.720
Stillbirth	12 (2%)	5 (1%)	0,220
Macrosomia (birthweight > 4,000 g)	9 (1.6%)	26 (4.5%)	0.03*
Preeclampsia	11 (1.9%)	5 (0.9%)	0.131
PPROM	9 (1.6%)	1 (0.2%)	0.011*
PROM	69 (12%)	68 (11%)	0.927
SGA (< 10th percentile)	10 (1.7%)	21(3.6%)	0.312
Low birthweight (< 2,500 g)	74 (12%)	29 (5.8%)	< 0.001*
Postpartum hemorrhage	15 (2.6%)	10 (2%)	0.546

Abbreviations: PROM, premature rupture of membrane; PPROM, preterm premature rupture of membrane; SGA, small for gestational age.

* *p* < 0.05, significant.

**Table 4:** Neonatal characteristics

	Refugee group	Control group	*p*-value
	(*n* = 576)	(*n* = 576)	
Birthweight (g) (mean ± SD)	3,013 ± 520	3,199 ± 500	< 0.001*
Height (cm) (mean ± SD)	49.1 ± 3.2	50.5 ± 19.3	0.092
Head circumference (cm) (mean ± SD)	34.3 ± 1.7	34.5 ± 1.4	< 0.001*
Apgar score n (%)			
< 7 at 1th minute	39 (6.8%)	21 (4.2%)	0.044*
< 7 at 5th minute	12 (2%)	2 (0.4%)	0.015*
NICU admission (n/ %)	54 (5.9%)	35 (6.1%)	0.115
Fetal sex (n/ %)			0.135
Female	278 (48%)	283 (49%)	
Male	298 (51%)	293 (51%)	

Abbreviation: NICU, requirement of neonatal intensive care unit.

* *p* < 0.05, significant.

A binary logistic regression was performed to test the main effects of nationality, adolescent pregnancy, parity, low education level, WGDP, and anemia on the likelihood that women have preterm birth, PPROM, and low birthweight. As shown in Table 5, low education level (OR = 1.7, 95% CI = 0.5–0.1), and WGDP (OR = 1.7, 95% CI = 0.5–0.1) in pregnancy were significant indicators for preterm birth/PPROM, and low birthweight adjusted for age, parity, nationality, and anemia.

**Table 5:** Logistic regression analysis of independent risk factors for preterm birth/PPROM, and Low birth weight

	Wald	OR (95% CI)	*p*-value
Adolescent pregnancy (age < 21)	0.92	1.1 (0.16–0.17)	0.226
Parity	0.2	0.9 (0.07–0,1)	0.478
Low education level (uneducated)	12.49	1.7 (0.5–0.1)	< 0.001*
WDPG	13.89	1.7 (0.5–0.1)	< 0.001*
Nationality (Syrian)	2.19	1.3 (0.2–0.1)	0.138
Anemia	0.16	1 (0.06–0.1)	0.547

Abbreviations: CI, confidence interval; OR, odds ratio; WGDP, weight gain during pregnancy.

* *p* < 0.05 is considered statistically significant.

## Discussion

In the present study, we analyzed and compared the obstetric and neonatal outcomes of Syrian refugees and ethnic Turkish women. First, the proportions of adolescent pregnancies—pregnancies at < 18 years—and mean gravidity were significantly higher among the Syrian refugees. Additionally, mean age, gestational age at delivery, WGPD, the proportion of uneducated women, and rate of antenatal follow-up were significantly lower in the refugee group. Secondly, our study showed that Syrian refugees were at increased risk of certain adverse obstetric outcomes, including preterm delivery, PPROM, and lower birth weight.

Previous refugee studies done in Turkey showed that Syrian pregnant women were younger than Turkish patients, and their mean age ranged from 23 to 25 years. Similarly, we found that mean age in our refugee group was 23 years.^13^ ^14^ Also, adolescent pregnancies have been reported to occur at a high rate among Syrian refugees. One third of the Syrian patients in this study were adolescents, and 10% of all pregnancies were in women under the age of 18 years. The proportion of adolescent pregnancies under 19 years of age was significantly higher among the Syrian patients (10% versus 1%, *p* < 0.001). A study by Cetorelli et al^15^ showed that adolescent fertility increased by over 30% soon after the onset of the Iraq war, while total fertility remained stable. They posited that adolescent girls may have been forced into marriage due to the lack of alternatives and a belief of their families that early marriage was the best way to protect the girls during this turbulent period.^15^

Adolescent pregnancies are riskier for both the mothers and their newborns, in terms of the rates of preterm delivery, PPROM, low birth weight (< 2,500 g), and anemia.^16^ ^17^ Similarly, we found that our Syrian refugees had higher rates of preterm delivery rates, PPROM, low birth weight, and anemia. Additionally, according to our data, the birth weight, head circumference, and rate of Apgar score < 7 at the 1th and 5th minutes of the neonates born to Syrian refugees were lower than those of neonates born to Turkish women. The difference of birth weight between the two groups cannot be statistically significant for term fetuses with birth weights between 10 and 90 percentile. Nevertheless, the number of infants with birth weights under 2,500 g was significantly higher in the refugee group. Low birth weight is correlated with high mortality and morbidity in the neonatal period.

In a systematic review, Villalonga-Olives et al^18^ evaluated pregnancy and birth outcomes among immigrant women in the US and Europe and demonstrated that the prevalence of low birth weight among migrants varied by host country, as well as the composition of migrants to different regions. This can be explained by the reduced WGDP, lack of routine antenatal follow-up, nutritional deficiencies, and insufficient iron supplementation among Syrian refugees. In logistic regression analysis, we found that only low education level and lower WGDP were more likely to be at risk of preterm birth/PPROM and low birthweight. For pregnant refugee women, malnutrition may pose a challenge due to insufficient access to food in war-torn environments and refugee camps. Ongoing nutritional support, counseling, and early intervention are important to promote healthy nutrition choices during pregnancy. The health of pregnant refugees and their forthcoming babies is mostly associated with the same factors as in native populations, but there are some additional factors that apply specifically to pregnant refugees: income level in the country of origin, fear of persecution, exposure to trauma, the asylum process, social background, employment status, living conditions, acculturation, and language-related factors.^19^ Posttraumatic stress disorder (PTSD) has been shown to be more frequent among asylum seekers and refugees,^20^ ^21^ and this can lead to adverse pregnancy outcomes, such as preterm birth and low birth weight,^22^ ^23^ due to changes in stress hormones.

Prenatal care is an important component of basic maternal healthcare; mothers should receive at least four antenatal visits.^24^ Of the Syrian refugees in our study, 67% did not receive antenatal care; this percentage was only 14% among the Turkish women. Furthermore, only 15% of the Syrian refugees underwent double/triple prenatal tests and a GDM screening test. Erenel et al^14^ studied antenatal care among Syrian refugees in Turkey and found this rate to be 41.3%. Many factors may be associated with decreased antenatal care. Although the “open-gate” policy of Turkey has saved the lives of many people fleeing persecution, the cost of health services has reached a high level. According to data from the Turkish Ministry of Health in 2015, 35,000 Syrians gave birth in Turkey.^25^ Reproductive health counseling and contraceptive materials are supplied by family planning services free of charge. Illiteracy can be an obstacle to antenatal care services among Syrian refugees. In agreement with this information, we demonstrated that half of our Syrian refugees were uneducated. Demirci et al^13^ showed that the percentage of women reporting illiteracy was higher among Syrian refugees, at 67.2%.^10^ Communication between healthcare professionals and pregnant Syrian women is a major problem.^26^ Although the Turkish government allocates translators to health services, their number is insufficient. Therefore, leaflets on safe motherhood and pre and postpartum care practices are written in Arabic and disseminated to refugees. Linguistic diversity and cultural barriers can negatively affect treatments and use of an interpreter may decrease the probability of poor obstetric outcomes. Several studies have been done on maternal outcomes among refugees of different ethnicities. However, there have been few reports on maternal outcomes among the Syrian refugee population of Turkey.

In terms of birth outcomes, we found that the primary C-section rate was higher among Turkish women than among Syrian refugees (21% versus 16%, *p* = 0.034). This may be related to differences between the nations. In another study done by Alnuaimi et al,^27^ they found that Syrian refugee mothers had a significant increase in the rate of overall C-section when compared with their Jordanian counterparts (36% versus 21%, *p* < 0.001). They explained that the most common reason for the high cesarean rates may be related to the high reported rate of previous C-section in Syria (20%). But in our study, this rate was found to be as low as 15%, and there was no statistical difference between the groups. Similarly, two previous studies done in Turkey demonstrated lower C-section rates among nulliparous Syrian patients;^13^ ^14^ they reported that their control groups were more likely to show the complications of GDM and preeclampsia. Our hospital is a reference hospital for high-risk pregnancies and older maternal age. The rate of fetal macrosomia was significantly higher among the pregnant Turkish women. However, we could not calculate the prevalence of GDM in the refugee group because few of the Syrian patients underwent a GDM screening test; thus, this parameter could not be compared between the groups. Also, the need for labor induction was significantly lower among the Syrian women (*p* < 0.001). The proportion of emergency births was higher among Syrian refugees than the Turkish control group (20% versus 4%, *p* < 0.001). One reason for this could be the preference for oxytocin, instead of dinoprostone, seen among the Syrian women for the purpose of labor induction. It is probable that they had greater cervical dilatation and higher Bishop scores at the time of hospital admission.

## Conclusion

In conclusion, obstetric outcomes among the Syrian refugees differed significantly from those of the Turkish women. Several different factors may influence these findings; thus, refugee women would benefit from more targeted care during pregnancy and childbirth.
